# Associations between source of information about sex and sexual health outcomes in Britain: findings from the third National Survey of Sexual Attitudes and Lifestyles (Natsal-3)

**DOI:** 10.1136/bmjopen-2015-007837

**Published:** 2015-03-04

**Authors:** Wendy Macdowall, Kyle G Jones, Clare Tanton, Soazig Clifton, Andrew J Copas, Catherine H Mercer, Melissa J Palmer, Ruth Lewis, Jessica Datta, Kirstin R Mitchell, Nigel Field, Pam Sonnenberg, Anne M Johnson, Kaye Wellings

**Affiliations:** 1 Department of Social and Environmental Health Research, Centre for Sexual and Reproductive Health Research, London School of Hygiene and Tropical Medicine, London, UK; 2 Research Department of Infection and Population Health, University College London, London, UK; 3 NatCen Social Research, London, UK

**Keywords:** sex eduction, first intercourse, sexual health, cross-sectional survey

## Abstract

**Objectives:**

To examine variation in source of information about sexual matters by sociodemographic factors, and associations with sexual behaviours and outcomes.

**Design:**

Cross-sectional probability sample survey.

**Setting:**

British general population.

**Participants:**

3408 men and women, aged 17–24 years, interviewed from 2010–2012 for third National Survey of Sexual Attitudes and Lifestyles.

**Main outcome measures:**

Main source of information (school, a parent, other); age and circumstances of first heterosexual intercourse; unsafe sex and distress about sex in past year; experience of sexually transmitted infection (STI) diagnoses, non-volitional sex or abortion (women only) ever.

**Results:**

Citing school was associated with younger age, higher educational level and having lived with both parents. Citing a parent was associated, in women, with lower educational level and having lived with one parent. Relative to other sources, citing school was associated with older age at first sex (adjusted HR 0.73 (95% CI 0.65 to 0.83) men, 0.73 (0.65 to 0.82) women), lower likelihood of unsafe sex (adjusted OR 0.58 (0.44 to 0.77) men, 0.69 (0.52 to 0.91) women) and previous STI diagnosis (0.55 (0.33 to 0.91) men, 0.58 (0.43 to 0.80) women) and, in women, with lower likelihood of lack of sexual competence at first sex; and experience of non-volitional sex, abortion and distress about sex. Citing a parent was associated with lower likelihood of unsafe sex (0.53 (0.28 to 1.00) men; 0.69 (0.48 to 0.99) women) and, in women, previous STI diagnosis.

**Conclusions:**

Gaining information mainly from school was associated with lower reporting of a range of negative sexual health outcomes, particularly among women. Gaining information mainly from a parent was associated with some of these, but fewer cited parents as a primary source. The findings emphasise the benefit of school and parents providing information about sexual matters and argue for a stronger focus on the needs of men.

**Strengths and limitations of this study**

- The size and nature of the sample which was selected using probability sampling and so is broadly representative of the British population.
- The range of demographic and sexual health factors included in the survey that allow examination of how learning about sex varies by markers social inequality, and examination of associations between sources of information and a broader range of sexual health factors than has been investigated before.
- Although the sample reflects the wider British population, in terms of demographic characteristics, it is possible that individuals who agree to take part in a sexual behaviour survey may differ from those who do not.
- As an observational, cross-sectional study, we are not able to infer causality or for some outcomes, temporality.
- Recall of the experience of learning about sexual matters may be recast with time, though we limited our analysis to individuals aged 17–24 years in order to minimise the potential bias associated with this.

## Introduction

Over recent decades, school lessons have risen in prominence as the main source of information about sexual matters for both boys and girls in Britain.^1^ Although guidance exists,^2^ ^3^ there is no statutory programme of study for sex and relationship education (SRE) beyond that included in the National Curriculum Science^i^ 2 and there are concerns about variations in the content and quality of provision.^4^

Disparities in the provision of SRE may be a mediating factor in social inequalities observed in sexual health.^5^ Earlier first intercourse (before 16 years)—a known risk factor for subsequent negative sexual health outcomes—occurs more commonly among those of lower educational level and lower socioeconomic status.^6^ Sexually transmitted infections (STIs) disproportionately affect those living in more deprived areas^7^ and certain ethnic minority groups;^8^ among women, unplanned pregnancies are associated with lower educational levels^9^ and experience of non-volitional sex with living in more deprived areas.^10^

Evidence suggests that school-based sex education delays the onset of sexual activity, and increases condom and contraceptive use among those already sexually active.^11–13^ Opponents of school SRE tend to focus on the argument that teaching young people about sexual matters should be the responsibility of parents.^14^ However, few young people cite a parent as a source of information about sex^1^ and the evidence of a positive relationship between provision of sex education by parents and sexual behaviour and sexual health outcomes is mixed.^15–17^

Existing research has mainly focused on whether school SRE or parental communication about sex improves biomedical aspects of sexual health^11^ and thus, reflects the framing of sexual health predominantly in terms of the prevention of adverse sexual health outcomes, such as STIs and unintended conceptions. Pleas have, however, been made for the adoption of a broader concept of sexual health, one that includes outcomes relating to the quality and consensuality of sexual experience, not only as risk factors for outcomes such as STIs and unintended conception, but as important ends in themselves.^18^

The National Survey of Sexual Attitudes and Lifestyles (Natsal) is a large and comprehensive probability sample survey of the British population. Findings from the first survey, conducted in 1990–1991,^19^ ^20^ and the second, in 1999–2001,^21–24^ have been extensively used to inform sexual and reproductive health policy in Britain.^25–27^ We use data from the third survey (Natsal-3), in 2010–2012, to explore how sources of information about sexual matters vary by sociodemographic factors; we examine associations between these sources and a wider range of sexual health outcomes than has previously been explored.

## Methods

Natsal-3 is a multistage, clustered and stratified probability sample survey of 15 162 men and women aged 16–74 years, resident in Britain. Postcode sectors were primary sampling units; addresses within them were selected at the second stage and one eligible adult was randomly selected at the final stage. To allow detailed exploration of behaviours in the age group at highest risk of certain sexual health outcomes, individuals aged 16–34 were oversampled. Addresses were randomly allocated to either the core sample (in which all individuals aged 16–74 were eligible) or one of two boost samples (boost 1, in which one person aged 16–34 years was selected or boost 2, in which one person aged 16–29 years was selected). The data was weighted to adjust for the unequal probabilities of selection and non-response. Participants were interviewed between September 2010 and August 2012 using computer-assisted personal interviewing (CAPI), including a computer-assisted self-interview for the more sensitive questions. The response rate was 57.7% for the whole sample, 64.8% for boost 1 and 67.3% for boost 2. Further details of the methods are described elsewhere.^28^

Questions relating to learning about sex were asked face-to-face in the CAPI section of the questionnaire (available at natsal.ac.uk). Participants were asked, “When you were growing up, in which of the ways listed on this card did you learn about sexual matters?” and “From which did you learn the most?” In response to the latter, they were requested to select one main source. In this paper, we categorised main source of sex education as: school lessons, provision by a parent and ‘other’ sources (which included first boyfriend/girlfriend/sexual partner, peers, siblings, internet sources, pornography, media sources, health professionals and other). All analyses were restricted to those aged 17–24 years at interview (1509 men and 1899 women). Participants aged 16 years were excluded as they could not be ascribed an educational level.

We examined the associations between a range of sociodemographic factors and main source of information about sexual matters by gender, including: age at interview; educational level; religiosity (a combined variable of religion considered ‘very’ or ‘fairly’ important and attendance at religious services at least once every two weeks); family structure (whether lived with both, one or neither natural parent(s) ‘more or less continuously’ until age 14); area-level deprivation (measured using the Index of Multiple Deprivation, a multidimensional measure combining income, employment, health, education, access to housing and services, crime and living environment^29^); type of school attended (mixed or single sex); and country of residence (England, Scotland or Wales).

We then examined associations between the main source of information about sexual matters, and key sexual behaviours and outcomes. These included: first heterosexual intercourse before age 16 years; lack of sexual competence at first heterosexual intercourse (defined as having not met the following self-reported four criteria: both partners ‘equally willing’, use of reliable contraception, autonomy of decision—not due to peer pressure, drunkenness or drugs—and occurrence at the perceived ‘right time’^22^); unsafe sex in the past year (defined as no condom used at the first occasion of sex with a new partner in the past year); distress about sex life in the past year (based on agreement with the statement: “I feel distressed or worried about my sex life”); and ever had an experience of STI diagnosis, non-volitional sex and for women, abortion. A composite variable of ‘overall sexual health’ was constructed and participants were coded as having good overall sexual health if they did not report distress or worry about sex life in the past year, or ever having had experience of an STI diagnosis, non-volitional sex or (for women only) abortion.

We performed all analyses using the survey commands in Stata V.13.1,^30^ which account for the weighting, clustering and stratification of the Natsal-3 data. We assessed the association between sociodemographic factors and the primary source of information among participants, aged 17–24 years, using univariate logistic regressions. We used survival analysis methods to estimate the distribution of age at first heterosexual intercourse by primary source of information about sexual matters, censoring those who had not yet had sex at their age at interview. We conducted proportional hazards regression to calculate hazard ratios (HRs) adjusting for year of birth, educational level and family structure to represent the effect of primary source of information on the rate of first heterosexual intercourse.

We examined the associations between reporting school lessons, a parent or an ‘other’ main source of information, and sexual behaviours and sexual health outcomes in multivariable logistic regression. In the multivariable analysis, we ran two models which adjusted for those sociodemographic variables found to be significantly associated with main source of sex education in our univariate analysis. In the first, we included all participants aged 17–24 years and adjusted for age, educational level and family structure. In the second, we restricted the analysis to sexually experienced individuals aged 17–24 years and adjusted for educational level, family structure, age at first intercourse and number of years sexually active. The latter approach was taken to assess the association between main source of information and sexual health outcomes independently of age at first sex; this informally represents the ‘direct effect’ of source of sex education on outcomes aside from any effect mediated through age at first intercourse.

## Results

### Main source of information and demographic factors

Overall, similar proportions of men and women reported lessons at school as their main source of information about sexual matters (37.5% (34.8% to 40.2%) and 39.5% (37.0% to 42.0%), respectively). Considerably fewer participants cited a parent and here there was a gender difference; the proportion of women doing so being twice that of men (14.6% (12.9% to 16.4%) and 7.3% (5.9% to 9.0%), respectively). The remainder—just over half of men (55.3% (52.4% to 58.1%)) and just under half of women (46.0% (43.4% to 48.5%))—reported their main source as being other than school or a parent (table 1).

**Table 1:** Main source of information about sexual matters by sociodemographic factors, men and women

	Other*					School					A parent					Denominators‡
	Per cent	95% CI	OR	95% CI	p Value†	Per cent	95% CI	OR	95% CI	p Value†	Per cent	95% CI	OR	95% CI	p Value†	
*All men, 17–24 years old*	55.3	(52.4 to 58.1)	–	–	–	37.5	(34.8 to 40.2)	–	–	–	7.3	(5.9 to 9.0)	–	–	–	1509, 1108
Age at interview					0.0041					0.0001					0.0610	
17–20	51.0	(47.1 to 55.0)	1.00			43.1	(39.2 to 47.0)	1.00			5.9	(4.5 to 7.8)	1.00			825, 564
21–24	59.7	(55.4 to 63.8)	1.42	(1.12 to 1.80)		31.7	(28.0 to 35.6)	0.61	(0.48 to 0.78)		8.7	(6.4 to 11.6)	1.51	(0.98 to 2.32)		684, 544
Academic qualifications					0.0031					0.0037					0.1919	
Studying for/attained further academic qualifications	51.7	(48.1 to 55.3)	1.00			41.5	(38.1 to 44.9)	1.00			6.9	(5.3 to 8.9)	1.00			957, 716
Academic qualifications typically gained at age 16	59.1	(53.5 to 64.4)	1.35	(1.03 to 1.77)		33.0	(28.2 to 38.2)	0.69	(0.53 to 0.91)		8.0	(5.2 to 12.1)	1.18	(0.69 to 2.00)		416, 285
No academic qualifications	72.3	(59.4 to 82.3)	2.44	(1.35 to 4.41)		24.5	(14.7 to 37.8)	0.46	(0.24 to 0.87)		3.2	(1.3 to 7.9)	0.45	(0.17 to 1.22)		96, 71
Religion important and practiced regularly					0.8036					0.3190					0.2022	
No	55.4	(52.4 to 58.3)	1.00			37.1	(34.3 to 39.9)	1.00			7.6	(6.1 to 9.4)	1.00			1391, 1013
Yes	54.1	(44.5 to 63.5)	0.95	(0.64 to 1.42)		41.9	(32.9 to 51.5)	1.22	(0.82 to 1.83)		4.0	(1.5 to 10.4)	0.51	(0.18 to 1.44)		118, 94
Family background until age 14					0.0200					0.0241					0.2003	
Lived with both natural parents	53.7	(50.3 to 57.1)	1.00			39.4	(36.3 to 42.7)	1.00			6.8	(5.3 to 8.8)	1.00			1032, 799
Lived with one natural parent	57.5	(52.2 to 62.6)	1.17	(0.91 to 1.49)		33.4	(28.7 to 38.3)	0.77	(0.60 to 0.98)		9.2	(6.2 to 13.3)	1.37	(0.84 to 2.24)		436, 283
Lived with neither	78.2	(60.2 to 89.5)	3.09	(1.34 to 7.13)		21.8	(10.5 to 39.8)	0.43	(0.19 to 0.99)		0.0	–	NA	–		41, 26
Region					0.7919					0.8044					0.2040	
England	55.2	(52.1 to 58.2)	1.00			37.7	(34.8 to 40.7)	1.00			7.1	(5.6 to 9.0)	1.00			1300, 954
Wales	52.8	(42.4 to 62.8)	0.91	(0.59 to 1.39)		34.6	(26.4 to 43.9)	0.88	(0.58 to 1.31)		12.6	(6.3 to 23.8)	1.89	(0.85 to 4.19)		90, 59
Scotland	57.7	(47.4 to 67.4)	1.11	(0.72 to 1.70)		36.8	(27.9 to 46.7)	0.96	(0.63 to 1.47)		5.5	(2.7 to 10.8)	0.76	(0.35 to 1.64)		119, 95
Quintiles of multiple deprivation					0.4910					0.8631					0.3730	
1 (least deprived)	52.1	(45.0 to 59.2)	1.00			40.6	(33.8 to 47.7)	1.00			7.3	(4.1 to 12.7)	1.00			269, 190
2	54.8	(48.7 to 60.8)	1.11	(0.76 to 1.64)		36.6	(30.7 to 42.9)	0.84	(0.56 to 1.27)		8.6	(5.4 to 13.4)	1.19	(0.55 to 2.61)		287, 212
3	51.6	(44.9 to 58.2)	0.98	(0.67 to 1.43)		38.7	(32.5 to 45.3)	0.93	(0.62 to 1.37)		9.7	(6.4 to 14.4)	1.37	(0.64 to 2.91)		279, 197
4	57.9	(51.5 to 64.0)	1.26	(0.86 to 1.86)		36.0	(30.0 to 42.4)	0.82	(0.55 to 1.23)		6.1	(3.7 to 9.9)	0.83	(0.37 to 1.85)		324, 260
5 (most deprived)	58.3	(52.2 to 64.1)	1.28	(0.88 to 1.87)		36.4	(31.1 to 42.1)	0.84	(0.58 to 1.22)		5.3	(3.1 to 8.8)	0.71	(0.31 to 1.61)		350, 248
Last school attended					0.7647					0.5731					0.6364	
Mixed school	55.1	(52.1 to 58.1)	1.00			37.7	(34.9 to 40.6)	1.00			7.2	(5.7 to 8.9)	1.00			1387, 1015
Single sex school	56.7	(46.3 to 66.5)	1.07	(0.70 to 1.64)		34.8	(25.8 to 45.0)	0.88	(0.57 to 1.37)		8.5	(4.2 to 16.3)	1.20	(0.56 to 2.59)		121, 93
*All women, 17–24 years old*	46.0	(43.4 to 48.5)	–	–	–	39.5	(37.0 to 42.0)	–	–	–	14.6	(12.9 to 16.4)	–	–	–	1899, 1088
Age at interview					0.0041					0.0052					0.8286	
17–20	42.2	(38.8 to 45.7)	1.00			43.0	(39.6 to 46.5)	1.00			14.7	(12.5 to 17.3)	1.00			968, 531
21–24	49.5	(45.9 to 53.2)	1.34	(1.10 to 1.64)		36.1	(32.7 to 39.6)	0.75	(0.61 to 0.92)		14.4	(12.1 to 17.0)	0.97	(0.74 to 1.28)		931, 557
Academic qualifications					0.7822					0.0628					0.0307	
Studying for/attained further academic qualifications	45.6	(42.6 to 48.7)	1.00			40.6	(37.6 to 43.7)	1.00			13.7	(11.7 to 16.1)	1.00			1199, 720
Academic qualifications typically gained at age 16	44.1	(39.3 to 48.9)	0.94	(0.75 to 1.18)		40.4	(35.6 to 45.4)	0.99	(0.78 to 1.26)		15.6	(12.5 to 19.1)	1.16	(0.84 to 1.59)		518, 266
No academic qualifications	47.3	(38.1 to 56.7)	1.07	(0.73 to 1.57)		29.5	(21.8 to 38.6)	0.61	(0.41 to 0.92)		23.2	(16.0 to 32.4)	1.90	(1.17 to 3.08)		133, 64
Religion important and practiced regularly					0.8944					0.8546					0.6811	
No	46.0	(43.4 to 48.6)	1.00			39.6	(37.0 to 42.2)	1.00			14.4	(12.7 to 16.3)	1.00			1757, 991
Yes	45.3	(35.7 to 55.3)	0.97	(0.65 to 1.46)		38.7	(30.1 to 48.1)	0.96	(0.65 to 1.43)		16.0	(9.8 to 25.0)	1.13	(0.63 to 2.02)		139, 96
Family background until age 14					0.9069					0.0360					0.0003	
Lived with both natural parents	46.3	(43.0 to 49.6)	1.00			41.6	(38.5 to 44.8)	1.00			12.1	(10.2 to 14.2)	1.00			1163, 709
Lived with one natural parent	45.1	(41.0 to 49.4)	0.95	(0.77 to 1.18)		35.1	(31.0 to 39.3)	0.76	(0.61 to 0.95)		19.8	(16.6 to 23.4)	1.79	(1.35 to 2.38)		676, 353
Lived with neither	44.9	(31.1 to 59.6)	0.95	(0.53 to 1.69)		44.5	(30.8 to 59.0)	1.12	(0.63 to 2.00)		10.6	(4.5 to 23.2)	0.86	(0.35 to 2.13)		59, 24
Region					0.8625					0.2827					0.2689	
England	45.8	(43.0 to 48.7)	1.00			39.8	(37.1 to 42.5)	1.00			14.4	(12.6 to 16.4)	1.00			1605, 938
Wales	48.7	(38.2 to 59.3)	1.12	(0.73 to 1.73)		31.6	(22.5 to 42.3)	0.70	(0.44 to 1.12)		19.7	(13.2 to 28.4)	1.46	(0.90 to 2.39)		114, 56
Scotland	45.5	(39.2 to 51.9)	0.98	(0.74 to 1.30)		41.4	(34.0 to 49.2)	1.07	(0.77 to 1.49)		13.2	(8.6 to 19.5)	0.90	(0.55 to 1.47)		180, 93
Quintiles of multiple deprivation					0.3180					0.8963					0.4657	
1 (least deprived)	42.1	(36.4 to 48.0)	1.00			40.8	(35.1 to 46.7)	1.00			17.1	(13.4 to 21.7)	1.00			319, 182
2	50.5	(44.7 to 56.3)	1.40	(1.01 to 1.94)		37.4	(31.9 to 43.3)	0.87	(0.62 to 1.21)		12.1	(8.7 to 16.5)	0.66	(0.42 to 1.06)		329, 185
3	45.1	(39.1 to 51.1)	1.13	(0.80 to 1.60)		39.3	(33.6 to 45.3)	0.94	(0.66 to 1.33)		15.7	(11.9 to 20.3)	0.90	(0.59 to 1.37)		357, 217
4	45.0	(39.5 to 50.5)	1.12	(0.81 to 1.56)		41.1	(35.6 to 46.8)	1.01	(0.72 to 1.42)		13.9	(10.6 to 18.1)	0.78	(0.51 to 1.20)		422, 250
5 (most deprived)	47.1	(41.8 to 52.5)	1.23	(0.89 to 1.69)		38.7	(33.5 to 44.2)	0.92	(0.66 to 1.28)		14.1	(10.8 to 18.2)	0.80	(0.52 to 1.21)		472, 253
Last school attended					0.1859					0.5724					0.2410	
Mixed school	45.2	(42.4 to 48.0)	1.00			39.9	(37.2 to 42.6)	1.00			15.0	(13.2 to 16.9)	1.00			1692, 954
Single sex school	51.1	(42.9 to 59.3)	1.27	(0.89 to 1.81)		37.3	(29.4 to 45.9)	0.90	(0.61 to 1.31)		11.6	(7.6 to 17.4)	0.75	(0.46 to 1.22)		203, 132

*Includes first boyfriend/girlfriend/sexual partner, peers, siblings, internet sources, pornography, media sources, health professionals and other.

†χ^2^ test of heterogeneity.

‡Denominators are unweighted, weighted.

The likelihood of citing school as a main source was higher among those of younger age; men and women aged 21–24 years were less likely to report school compared with those aged 17–20 years (table 1). It was also higher among men and women studying for or who had achieved qualifications post 16—and for women among those with qualifications typically gained at 16—as opposed to those with none and among those living with both natural parents as opposed to only one (and for men who lived with neither).

The likelihood of citing a parent as the main source was, in women, higher among those without qualifications compared with those with or likely to obtain them, and among those who lived with one natural parent as opposed to two or neither (table 1).

The likelihood of citing an ‘other’ main source of sex education was higher among those aged 21–24 than those aged 17–20. Among men, it was also higher among those with minimum or no qualifications, and those living with neither natural parent.

Main source of information was not associated with religiosity, area level deprivation, whether the school attended was mixed or single sex, or country of residence (table 1).

### Main source of information and sexual behaviour and outcomes

**Figure 1:**
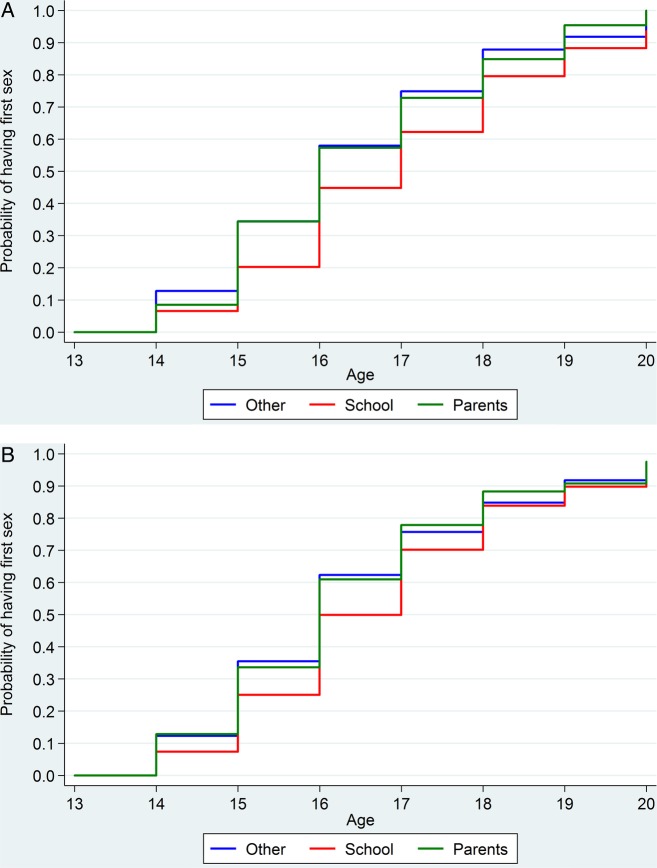
Kaplan-Meier estimates of the probability of having first heterosexual sex at, or before, each age by main source of information (A) men aged 17–24 and (B) women aged 17–24.

The survival analysis showed that after adjusting for age at interview, education and family structure, participants who reported school as their main source of sexual information had first intercourse at comparatively later ages than did those whose main source was ‘other’ (men who reported lessons from school had a HR of 0.73 (95% CI 0.65 to 0.83) for having first sex relative to men who reported an ‘other’ source, the corresponding ratio for women was 0.73 (0.65 to 0.82); figure 1A, B). No association was found between citing a parent as a main source and age at first intercourse. Note this regression analysis is informal because the assumption of proportional hazards is not met. Specifically, while citing school as main source of sex education is associated with a lower rate of having first sex relative to other sources at younger ages, it is associated with a higher rate at higher ages. By age 20 (more clearly among women) the proportion that has had sex seems unrelated to source of sex education.

Men for whom school was the main source of information were less likely than those reporting an ‘other’ main source to have had unsafe sex in the past year (OR 0.58 (95% CI 0.44 to 0.77)) or ever being diagnosed with an STI (0.55 (0.33 to 0.91); table 2). Among sexually experienced men, the association with unsafe sex in the past year remained strong (0.66 (0.49 to 0.89)), but was attenuated for ever being diagnosed with an STI (0.72 (0.43 to 1.22)).

**Table 2:** Sexual behaviours and outcomes by main source of information about sexual matters, men and women

	Of all men*							Of sexually experienced men†						
	Other		School		A parent		p Value‡	Other		School		A parent		p Value‡
First sex before age 16							<0.0001							0.0001
Per cent (95% CI)	35.9%	(32.4 to 39.6)	20.7%	(17.3 to 24.6)	40.3%	(29.7 to 51.9)		41.4%	(37.4 to 45.5)	26.8%	(22.5 to 31.7)	44.0%	(32.5 to 56.2)	
AOR (95% CI)	1.00		0.47	(0.35 to 0.64)	1.12	(0.69 to 1.82)		1.00		0.50	(0.37 to 0.69)	1.04	(0.63 to 1.73)	
Denominators	793, 583		548, 399		102, 73			695, 508		411, 309		89, 66		
Lack of sexual competence at first heterosexual sex§							0.7668							0.8434
Per cent (95% CI)	45.0%	(41.0 to 49.1)	40.5%	(34.6 to 46.6)	47.6%	(36.2 to 59.2)		45.0%	(41.0 to 49.1)	40.5%	(34.6 to 46.6)	47.6%	(36.2 to 59.2)	
AOR (95% CI)	1.00		0.93	(0.69 to 1.26)	1.13	(0.68 to 1.89)		1.00		0.98	(0.72 to 1.33)	1.15	(0.69 to 1.91)	
Denominators	689, 504		407, 306		89, 66			689, 504		407, 306		89, 66		
Unsafe sex¶							0.0003							0.0052
Per cent (95% CI)	28.5%	(25.1 to 32.1)	18.3%	(15.3 to 21.9)	18.9%	(11.5 to 29.5)		32.5%	(28.8 to 36.4)	23.6%	(19.6 to 28.2)	20.4%	(12.3 to 32.1)	
AOR (95% CI)	1.00		0.58	(0.44 to 0.77)	0.53	(0.28 to 1.00)		1.00		0.66	(0.49 to 0.89)	0.49	(0.25 to 0.95)	
Denominators	782, 574		554, 403		102, 73			675, 494		406, 305		87, 65		
Ever diagnosed with an STI							0.0553							0.3840
Per cent (95% CI)	9.5%	(7.6 to 11.9)	4.8%	(3.2 to 7.1)	8.2%	(3.6 to 17.7)		10.5%	(8.3 to 13.2)	6.3%	(4.2 to 9.3)	9.2%	(4.0 to 19.6)	
AOR (95% CI)	1.00		0.55	(0.33 to 0.91)	0.65	(0.24 to 1.75)		1.00		0.72	(0.43 to 1.22)	0.65	(0.23 to 1.85)	
Denominators	792, 582		560, 407		104, 74			684, 501		410, 308		89, 66		
Non-volitional sex, ever							0.6915							0.9410
Per cent (95% CI)	1.0%	(0.5 to 2.1)	0.7%	(0.2 to 1.9)	1.5%	(0.3 to 7.0)		1.2%	(0.6 to 2.5)	0.9%	(0.3 to 2.5)	1.7%	(0.3 to 7.8)	
AOR (95% CI)	1.00		0.65	(0.18 to 2.29)	1.35	(0.24 to 7.74)		1.00		0.94	(0.24 to 3.65)	1.31	(0.22 to 7.62)	
Denominators	777, 571		552, 401		103, 73			672 ,493		405, 304		88, 65		
Distressed/worried about sex life							0.3971							0.6328
Per cent (95% CI)	11.1%	(8.9 to 13.9)	10.8%	(8.2 to 14.0)	6.2%	(3.1 to 12.1)		9.6%	(7.4 to 12.3)	8.2%	(5.8 to 11.4)	6.2%	(2.9 to 12.4)	
AOR (95% CI)	1.00		0.95	(0.64 to 1.42)	0.59	(0.27 to 1.27)		1.00		0.84	(0.52 to 1.36)	0.71	(0.31 to 1.61)	
Denominators	762, 557		516, 378		102, 73			684, 501		410, 308		89, 66		
Overall sexual health**							0.3975							0.6780
Per cent (95% CI)	80.6%	(77.2 to 83.6)	84.2%	(80.5 to 87.3)	83.8%	(74.0 to 90.4)		81.4%	(78.0 to 84.4)	85.6%	(81.7 to 88.9)	82.8%	(72.1 to 90.0)	
AOR (95% CI)	1.00		1.22	(0.88 to 1.70)	1.34	(0.71 to 2.55)		1.00		1.18	(0.81 to 1.72)	1.17	(0.59 to 2.32)	
Denominators	745, 544		511, 374		101, 73			670, 490		405, 304		88, 65		

	Of all women*							Of sexually experienced women†						
	Other		School		A parent		p Value‡	Other		School		A parent		p Value‡
First sex before age 16							0.0001							0.0032
Per cent (95% CI)	33.4%	(30.0 to 37.0)	23.5%	(20.3 to 27.0)	33.6%	(27.8 to 40.0)		37.5%	(33.7–41.3)	30.0%	(26.1–34.3)	39.7%	(33.0–46.7)	
AOR (95% CI)	1.00		0.56	(0.43 to 0.72)	0.84	(0.62 to 1.14)		1.00		0.63	(0.48–0.82)	0.92	(0.66–1.27)	
Denominators	823, 462		706, 408		273, 151			748, 414		565, 321		235, 127		
Lack of sexual competence at first heterosexual sex§							0.0021							0.0131
Per cent (95% CI)	56.3%	(52.3 to 60.1)	47.1%	(42.6 to 51.6)	52.5%	(45.5 to 59.4)		56.3%	(52.3–60.1)	47.1%	(42.6–51.6)	52.5%	(45.5–59.4)	
AOR (95% CI)	1.00		0.65	(0.51 to 0.83)	0.75	(0.54 to 1.06)		1.00		0.70	(0.54–0.90)	0.75	(0.53–1.05)	
Denominators	741, 409		564, 320		234, 127			741, 409		564, 320		234, 127		
Unsafe sex¶							0.0141							0.1437
Per cent (95% CI)	26.1%	(22.9 to 29.5)	20.4%	(17.3 to 23.9)	21.4%	(16.5 to 27.3)		29.9%	(26.4–33.7)	26.3%	(22.4–30.5)	25.2%	(19.5–31.9)	
AOR (95% CI)	1.00		0.69	(0.52 to 0.91)	0.69	(0.48 to 0.99)		1.00		0.81	(0.60–1.08)	0.71	(0.48–1.04)	
Denominators	828, 465		714, 411		278, 155			736, 405		555, 315		234, 127		
Ever had an abortion							0.1014							0.2692
Per cent (95% CI)	10.5%	(8.4 to 13.0)	6.9%	(5.2 to 9.0)	7.7%	(5.1 to 11.4)		12.1%	(9.8–14.9)	8.5%	(6.4–11.2)	9.3%	(6.2–13.7)	
AOR (95% CI)	1.00		0.70	(0.47 to 1.04)	0.64	(0.38 to 1.08)		1.00		0.84	(0.56–1.27)	0.65	(0.38–1.11)	
Denominators	833, 470		723, 417		279, 155			740, 409		560, 319		235, 127		
Ever diagnosed with an STI							0.0004							0.0110
Per cent (95% CI)	20.8%	(17.8 to 24.1)	12.5%	(10.0 to 15.5)	13.8%	(10.1 to 18.4)		23.8%	(20.5–27.3)	16.2%	(13.0–20.0)	16.6%	(12.3–22.2)	
AOR (95% CI)	1.00		0.58	(0.43 to 0.80)	0.54	(0.36 to 0.82)		1.00		0.71	(0.50–0.99)	0.57	(0.38–0.86)	
Denominators	833, 468		717, 414		277, 154			740, 407		557, 317		234, 127		
Non-volitional sex, ever							0.0320							0.1325
Per cent (95% CI)	9.1%	(7.2 to 11.4)	5.9%	(4.3 to 8.1)	6.3%	(3.9 to 10.1)		10.5%	(8.3–13.2)	7.5%	(5.4–10.3)	7.2%	(4.4–11.6)	
AOR (95% CI)	1.00		0.61	(0.40 to 0.94)	0.58	(0.32 to 1.03)		1.00		0.76	(0.49–1.18)	0.55	(0.30–1.04)	
Denominators	813, 457		699, 404		272, 151			721, 397		538, 307		229, 124		
Distressed/worried about sex life							0.1757							0.0762
Per cent (95% CI)	11.4%	(9.1 to 14.2)	8.6%	(6.5 to 11.4)	11.3%	(7.5 to 16.8)		11.2%	(8.7–14.3)	7.3%	(5.2–10.0)	10.4%	(6.5–16.3)	
AOR (95% CI)	1.00		0.70	(0.47 to 1.03)	1.00	(0.58 to 1.70)		1.00		0.60	(0.38–0.94)	0.94	(0.52–1.68)	
Denominators	812, 453		666, 380		264, 145			739, 407		557, 317		234, 127		
Overall sexual health††							0.0001							0.0024
Per cent (95% CI)	60.1%	(56.3 to 63.8)	71.1%	(66.9 to 74.9)	68.5%	(61.9 to 74.4)		57.3%	(53.3–61.2)	68.5%	(63.9–72.8)	66.5%	(59.4–72.9)	
AOR (95% CI)	1.00		1.64	(1.28 to 2.10)	1.59	(1.12 to 2.26)		1.00		1.50	(1.14–1.96)	1.64	(1.13–2.38)	
Denominators	789, 440		641, 367		256, 141			717, 393		534, 306		228, 124		

Denominators are unweighted, weighted.

*OR is adjusted for age (continuous) educational level and family structure (binary variable of living with both natural parents/living with one/no parent).

†OR is adjusted for educational level, family structure, age at first sex (continuous) and years sexually active (continuous), except for “First sex before age 16” which is adjusted for educational level, family structure and age (continuous).

‡χ^2^ test of heterogeneity.

§Not met the following four criteria: both partners equally willing, use of reliable contraception, autonomy of decision, and that it happened at the ‘right time’. Excludes those who have not experienced heterosexual sex.

¶No condom used at the first occasion of sex with a new partner in the past year.

**Composite of: not experiencing non-volitional sex or STI diagnosis, and lack of distress about sex life in the past year.

††Composite of: not experiencing non-volitional sex, STI diagnosis, or abortion and lack of distress about sex life in the past year.

AOR, adjusted OR; STI, sexually transmitted infection.

Men citing a parent as their main source were less likely to have reported unsafe sex in the past year than those citing an ‘other’ main source (0.53 (0.28 to 1.00))—an association that remained in the analysis of sexually active men (0.49 (0.25 to 0.95)—but were no less likely to have been diagnosed with an STI (table 2).

Among women, reporting school as the main source of information was associated with a decreased likelihood of all the negative sexual health indicators examined in the multivariable analysis (table 2). Among sexually active women, the associations with lack of sexual competence at first intercourse (0.70 (0.54 to 0.90)), ever experiencing STI diagnosis (0.71 (0.50 to 0.99)), distress about sex life in the past year (0.60 (0.38 to 0.94)) and good ‘overall sexual health’ (1.50 (1.14–1.96)) remained, while those with unsafe sex in the past year (0.81 (0.60 to 1.08)), ever had an experience of abortion (0.84 (0.56 to 1.27)) and non-volitional sex (0.76 (0.49 to 1.18)) were in the same direction but were attenuated.

Among women, reporting a parent as the main source of information was also associated with a decreased likelihood of all the sexual health factors examined in the multivariable analysis, with the exception of sex before age 16 years and distress about sex life (table 2). The adjusted ORs were similar to those among women reporting school as a main source, though the CIs were slightly wider reflecting the smaller number of women reporting a parent. Among sexually active women citing a parent, the associations remained largely unchanged: sexual competence at first intercourse (0.75 (0.53 to 1.05)); unsafe sex (0.71 (0.48 to 1.04)); ever had an abortion (0.65 (0.38 to 1.11)); ever had an STI (0.57 (0.38 to 0.86)); non-volitional sex (0.55 (0.30 to 1.04)) and good ‘overall sexual health’ (1.59 (1.12 to 2.26)).

## Discussion

### Highlights

We found differences in the reporting of a range of sexual health indicators according to the main source of information about sexual matters. Receipt of information mainly from school, as opposed to other sources, was associated with lower reporting of a wide range of sexual health risk behaviours and outcomes. Receipt of information from a parent, as opposed to other sources, was associated with lower reporting of some but not all of these. For both school and parents, the range of outcomes where positive associations were found was wider in women than men.

### Strengths and weaknesses of the study

The strength of this study lies in the size and nature of the sample, which was selected using probability sampling and so is broadly representative of the British population. Another strength is the range of demographic and sexual health factors included in the survey that allow examination of both how learning about sex varies by markers social inequality and the associations between sources of information and a broader range of sexual health factors than has been investigated hitherto.

Several limitations, however, should be considered. Although the sample reflects the wider British population in terms of demographic characteristics, it is possible that individuals who agree to take part in a survey of this nature may differ from those who do not. Since this was an observational, cross-sectional study, we are not able to infer causality or for some outcomes, temporality. Relatedly, we cannot know whether some antecedent factor may predispose young people to seek higher academic achievement and to privilege school-based information. It is also important to note that the recall of the experience of learning about sexual matters may be recast with time, though we limited our analysis to individuals aged 17–24 years in order to minimise the potential bias associated with this. We must also acknowledge that a possible consequence of singling out one main source of sex education for the purpose of analysis is that the nuances of learning about sexual matters from multiple sources are lost.

### Strengths and weaknesses with respect to other studies and important differences in results

Our finding that school as the main source of sex education is associated with later age at first sex is consistent with that from other observational and intervention studies.^11^ ^13^ ^31^ As may be expected, associations with lower reporting of some of the sexual health factors we explored (for men, ever had diagnosis of an STI; and for women, unsafe sex in the past year and ever had experience of abortion or non-volitional sex) appear to be operating through later age at first intercourse. More surprising—and in contrast to research that has taken a similar approach elsewhere^31^—is the number of associations that remain after adjusting for age at first sex, years sexually active, educational level and family structure (for men a lower likelihood of having unsafe sex in the past year, and for women a lower likelihood of first sex being defined as lacking sexual competence, ever had diagnosis of an STI and distress about sex the past year), which suggests that school-based sex education is associated with additional benefit independent of that relating to later age at first sex.

As noted above, it has been suggested that variations in the provision of SRE may be a mediating factor in social inequalities observed in sexual health.^5^ Unlike researchers from the USA,^32^ we did not find area (neighbourhood) level deprivation to be associated with reporting school as a main source of sex education, though neighbourhood-level deprivation at the time of interview may have been different from that when growing up. We did, however, find school as a main source to be associated with educational level. Participants who had no qualifications (and among men only those typically gained at 16 years) were less likely to report school as their main source. Multiple, possibly inter-related, factors may help to explain this association. The Office for Standards in Education (Ofsted) found a strong correlation between a school's scores for performance generally and SRE, specifically.^4^ So it could be argued that ‘good’ SRE is an indicator of a ‘good’ school; one that better fosters the educational *and* personal and social development of young people. It has also been suggested that young people with lower psychosocial well-being do less well at school and are less engaged,^33^ traits which are both associated with increased risk of negative sexual health outcomes.^6^ ^9^ ^34^ Those ‘missing out’ on school-based SRE may be less of a concern in policy terms if they instead report a parent; indeed among women, those with no academic qualifications were more likely to do so, but this was not the case for men.

Studies exploring the relationship between parental communication and age at first sex have produced somewhat equivocal findings.^16^ ^17^ Some have suggested that parents may initiate or intensify communication about sexual matters once they think their children have become sexually active.^35^ This may explain the absence of an association between parents as a main source of information and later age at first intercourse. We did, however, see positive associations with other sexual health outcomes, notably safe sex. There is evidence that wider aspects of parenting, including good communication generally, parental monitoring and family ‘connectedness’ are positively associated with sexual health outcomes^16^ and that parents may wield an effect through their influence on risk behaviours, such as alcohol use^16^ ^17^ and/or by moderating peer pressure.^35^ As such, an exclusive focus on communication about sex may serve to underestimate the role of parents. However, the complex interplay of individual and family-related factors and their relative contribution to sexual health outcomes is poorly understood.

### Meaning of the study, possible explanations and implications for clinicians and policy makers

We found learning about sex mainly from school to be associated with the ‘stalwarts’ of sex education (age at first intercourse, safe sex and STIs) in men and women. Our finding that receipt of information mainly from school was associated with a wider number of sexual behaviours and outcomes among women than men has implications for policy and practice, and may be seen to warrant greater attention to the broader framing of sexual health in sex education, particularly for men. It has been suggested that sex education is overly focused on ‘girls’ issues’ (the so called ‘three Ps’: periods, pills and pregnancy) (Emmersen L, personal communication) and it is important that “issues such as relationships, consent, contraception and infections, are considered from a young man's perspective.”^36^ According to our study, men are also less likely than women to report a parent as a main source of sex education and as with school, doing so is associated with fewer positive outcomes than in women.

### Unanswered questions and future research

More nuanced research into the content, context and mode of delivery of sex education by both school and parents is needed. Also needed is longitudinal research to explore temporality in relation to learning about sex and sexual trajectories along with further intervention research, specifically exploring how best to meet the needs of young men and support parents in communicating about sexual matters in a timely manner. Multifaceted research exploring the relative contribution of different factors at play (including those related to community, school, family, peers and partners) and how they interact to mediate and/or moderate risk would be an important contribution to our understanding about how young people learn about sex and navigate early sexual experiences.

## Conclusion

Our findings emphasise the benefit of school and parents providing information about sexual matters and argue for a stronger focus on the needs of men. Parents, in particular, need to recognise their role, which is important not just in relaying information about sexual matters but also, more generally, in moderating risks faced by young people.

## Footnotes

i At the time of writing, a Government Education Select Committee was holding an inquiry into Personal, Social, Health and Economic Education (PSHE), and SRE in schools addressing: whether PSHE ought to be statutory; whether the current accountability system is sufficient to ensure that schools focus on PSHE; the overall provision of SRE in schools and the quality of its teaching; whether recent steps to supplement the guidance on teaching about sex and relationships are adequate; and how the effectiveness of SRE should be measured [http://www.parliament.uk/business/committees/committees-a-z/commons-select/education-committee/inquiries/parliament-2010/pshe-and-sre-in-schools].
